# Machine Learning Based Classification of Deep Brain Stimulation Outcomes in a Rat Model of Binge Eating Using Ventral Striatal Oscillations

**DOI:** 10.3389/fpsyt.2018.00336

**Published:** 2018-08-03

**Authors:** Wilder T. Doucette, Lucas Dwiel, Jared E. Boyce, Amanda A. Simon, Jibran Y. Khokhar, Alan I. Green

**Affiliations:** ^1^Department of Psychiatry, Geisel School of Medicine at Dartmouth, Hanover, NH, United States; ^2^The Dartmouth Clinical and Translational Science Institute, Dartmouth College, Hanover, NH, United States; ^3^Department of Psychological and Brain Sciences, Hanover, NH, United States; ^4^Department of Molecular and Systems Biology, Geisel School of Medicine at Dartmouth, Hanover, NH, United States; ^5^Department of Biomedical Sciences, University of Guelph, Guelph, ON, Canada

**Keywords:** binge eating, nucleus accumbens, deep brain stimulation (DBS), local field potential (LFP), machine learning applied to neuroscience

## Abstract

Neuromodulation-based interventions continue to be evaluated across an array of appetitive disorders but broader implementation of these approaches remains limited due to variable treatment outcomes. We hypothesize that individual variation in treatment outcomes may be linked to differences in the networks underlying these disorders. Here, Sprague-Dawley rats received deep brain stimulation separately within each nucleus accumbens (NAc) sub-region (core and shell) using a within-animal crossover design in a rat model of binge eating. Significant reductions in binge size were observed with stimulation of either target but with significant variation in effectiveness across individuals. When features of local field potentials (LFPs) recorded from the NAc were used to classify the pre-defined stimulation outcomes (response or non-response) from each rat using a machine-learning approach (lasso), stimulation outcomes could be classified with greater accuracy than expected by chance (effect sizes: core = 1.13, shell = 1.05). Further, these LFP features could be used to identify the best stimulation target for each animal (core vs. shell) with an effect size = 0.96. These data suggest that individual differences in underlying network activity may relate to the variable outcomes of circuit based interventions, and measures of network activity could have the potential to individually guide the selection of an optimal stimulation target to improve overall treatment response rates.

## Introduction

Brain stimulation has demonstrated the potential to improve symptoms in Parkinson's disease, depression and obsessive-compulsive disorder, yet highly variable treatment outcomes (especially common in psychiatric disorders) indicate that the full potential of brain stimulation is not being met (1–3). The majority of these studies evaluate the treatment outcomes of a single brain target despite pre-existing evidence supporting the potential of other stimulation targets (2–6). With these constraints, treatment outcome improvements have mostly been achieved to date through more stringent inclusion/exclusion criteria and improved precision in modulating the intended brain target (7–9). Another potential avenue to improve treatment outcomes for a specific disorder could be achieved through the personalization of target selection. This approach was pioneered by cancer biologists who used tumor immunoprofiling to personalize chemotherapy, and it remains unknown if personalization of target selection for neuromodulation-based treatments has a similar potential to improve treatment outcomes in neuropsychiatric diseases including disorders of appetitive behavior.

Clinical studies that used invasive or non-invasive stimulation in disorders of appetitive behavior (e.g., addiction, binge eating and obesity) have demonstrated the potential of targeting an array of different brain areas, but also demonstrated considerable treatment response heterogeneity across individuals (6, 10–14). The pre-clinical literature on deep brain stimulation (DBS), while also encouraging for appetitive disorders, reveals considerable outcome variation resulting from the targeting of different brain regions across studies. In addition, most studies report only group-based effects, masking the problem of variation across individuals (15–17).

In this study, we used an established rat model of binge eating to produce binge-like feeding behavior (18–20). Similar rodent models of binge eating have resulted in weight gain (20), compulsive feeding behavior (21, 22) and increased impulsivity (23) thus displaying traits commonly observed in appetitive disorders like substance use and binge eating disorder. It is important to acknowledge, however, that this is a pre-clinical approximation of the clinical condition, and many successful pharmacologic trials using this rodent/rat model have failed to translate clinically with the exception of lisdexamfetamine (24, 25). Using this pre-clinical model of binge eating, we have previously shown variation in individual rat outcomes receiving deep brain stimulation targeting the nucleus accumbens core with about 60% of rats displaying a significant reduction in binge size with stimulation (26). When non-invasive, repetitive transcranial magnetic stimulation was targeted to a related area of the reward circuit in patients with binge eating, the frequency of binges decreased in 18 of 28 subjects (~60%) (27). While the primary outcome in clinical and pre-clinical studies tend to be different (frequency of binges vs. size of binges), this rat model of binge eating could provide insight into stimulation outcome variability and provide a model to explore the potential feasibility and benefit of personalized target selection for stimulation-based interventions.

We theorize that individual variation in brain stimulation outcomes targeting a specific brain region may be linked to individual differences in the networks underpinning the symptom of interest (e.g., binge eating) (27). It follows that measures of relevant network activity could be used to predict brain stimulation outcomes at a given brain target or could be used to individualize the choice between potentially viable targets. This study evaluated the treatment efficacy of stimulation targeted to either the nucleus accumbens (NAc) core or shell, two regions with known differences in anatomical and functional connectivity and different functional roles across an array of reward-related behaviors (28, 29). This study replicated our previous treatment outcome variance with NAc core stimulation (26) and extended the results to assess whether similar variation in treatment outcomes occurs with NAc shell stimulation (previously reported by Halpern et al. to be effective in a mouse model of binge eating) (30, 31). We then determined whether a relationship existed between individual stimulation outcomes and either corresponding performance on reward-related behaviors, local field potential recordings from the NAc sub-regions or variation in electrode localization within each NAc sub-region.

## Materials and methods

### Animals and surgery

Male Sprague-Dawley rats were purchased from Charles River (*Shrewsbury, MA*) at 60 days of age and individually housed using a reverse 12 h light/dark schedule with house chow and water available *ad libitum*. Following habituation to the animal facility, rats were implanted with a custom electrode array that targeted both the NAc core and shell bilaterally, according to the following coordinates relative to bregma: 1.6 mm anterior; ± 1 and 2.5 mm lateral; and 7.6 mm ventral. Animals were excluded from analysis if later histological examination revealed electrode locations outside the NAc core or shell. All experiments were carried out in accordance with the NIH Guide for the Care and Use of Laboratory Animals (NIH Publications No. 80-23) revised in 1996 and approved by the Institutional Animal Care and Use Committee at Dartmouth College.

### Binge eating paradigm

**Figure 1:**
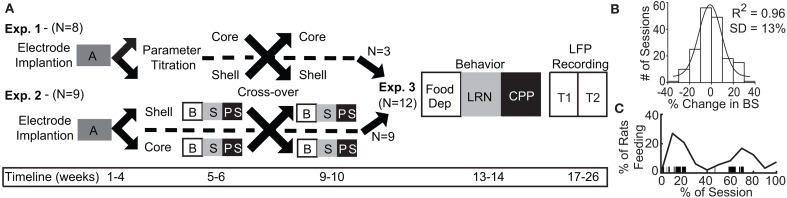
Experimental design and timeline with population data used to define significant change in binge size from baseline. **(A)** Experimental design for Experiments 1–3 with timeline shown at bottom. A, acquisition of stable binge size following chronic irregular limited access and randomization to initial stimulation target; B, baseline sessions; S, stimulation sessions; PS, post-stimulation sessions; Food Dep, food deprived binge session; LRN, locomotor response to novelty; CPP, conditioned place preference; LFP Recording, local field potential recording at two time points (T1 and T2). **(B)** Population baseline data (3 sessions per animal, *N* = 36 animals) was used to determine an *a priori* definition of a significant change from baseline binge size (BS). Distribution of binge size variance across baseline sessions was fit to a normal distribution with *R*^2^ fit shown (1 standard deviation [SD] = 13% change from baseline average). **(C)** The percentage of animals engaged in feeding behavior through a normalized binge session had a bimodal distribution. Vertical black lines under the curve provide an individual example of all of the feeding epochs from a single animal through a binge session.

Following recovery from surgery (~1 week), rats began a schedule of limited access to a palatable high-fat, high-sugar diet (“sweet-fat diet”), which contained 19% protein, 36.2% carbohydrates, and 44.8% fat by calories and 4.6 kcal/g (Teklad Diets 06415, *South Easton, MA*) as previously described (20). The sweet-fat diet was provided to the rats in addition to house chow and water within stimulation chambers for 2 h sessions during 4–5 sessions per week (irregular schedule). Following 16–20 sessions, the rats were consuming a stable and significant amount of sweet-fat food during each session [mean = 54% of their daily caloric intake ± 12% (1 standard deviation)]. This “binge-like” feeding has been shown to result in more significant weight gain than was observed with continuous access to the same diet—as is used in models of diet-induced obesity (20). Prior work has also demonstrated that chronic, irregular, limited access to palatable food can result in compulsive feeding behavior (21, 22) and increased impulsivity (23). Palatable sweet-fat and regular house chow consumption were measured during all limited access sessions. Video recordings were manually scored to assess the temporal dynamics of when feeding occurred during limited access sessions (Figure 1C).

### Stimulation

To deliver stimulation, a current-controlled stimulator (*PlexStim, Plexon, Plano, TX*) was used to generate a continuous train of biphasic pulses. The output of the stimulator (current and voltage) was verified visually for each rat before and after each stimulation session using a factory-calibrated oscilloscope (*TPS2002C, Tektronix, Beaverton, OR*). Stimulation was initiated immediately before animals had access to the sweet-fat food and turned off at the completion of the 2 h session.

### Overall design

Experiment 1 (*N* = 8 rats) was used to determine the optimal stimulation parameters to reduce binge size using our custom electrode arrays targeting the NAc core or shell. Experiment 2 (*N* = 9) used a crossover design in a separate cohort of rats to test DBS targeting the NAc core or shell with the optimized stimulation parameters identified in Experiment 1. Last, rats from Experiment 1 and 2 that had received the optimized stimulation parameters in both NAc targets and remained in good health (*N* = 12) continued on to Experiment 3 and underwent behavioral and electrophysiological characterization (Figure 1A).

## Experiment 1 – identifying optimal stimulation parameters

To identify the optimal stimulation parameters to alter feeding behavior, we tested an array of published stimulation intensities (range: 150–500 μA) and electrode contact configurations (monopolar vs. bipolar using our custom arrays within the targeted brain structures (NAc core and shell). These permutations alter the size and shape of the electric field and the resulting effect that stimulation has on binge eating. Rats were randomly divided into two groups for a crossover design with different initial stimulation targets (core or shell). Animals were then trained in the binge eating paradigm until a stable baseline of sweet-fat food intake was established (15–20 sessions over 3–4 weeks) before DBS sessions were initiated. Stimulation current was increased during each subsequent session, starting at 150 μA and progressing to 500 μA in a bipolar configuration (between two wires within the target, separated by ~1 mm in the dorsal-ventral plane), and then from 150 to 300 μA in a monopolar configuration (between one wire in the target and a skull screw over lambda). The rats then entered a period without DBS in which the effect of prior stimulation was allowed to washout before crossing over to DBS treatment of the other site. Following the washout and a return to baseline, we resumed stimulation in the other NAc target and the same titration of stimulation parameters was repeated at the second target of DBS across multiple sessions (Figure 1A).

## Experiment 2 – testing NAc core vs. shell stimulation using fixed stimulation parameters

Experiment 1 was designed to identify stimulation parameters that were similarly effective in either the NAc core or shell—bipolar stimulation at 300 μA or monopolar stimulation at 200 μA. We elected to use monopolar stimulation (biphasic, 90 μs pulse width, 130 Hz, 200 μA) as it produced a lower charge density at the electrode surface, which decreases the probability of neuronal injury (32). In a new cohort of rats, (*N* = 9) electrodes were implanted and rats were randomized to receive initial stimulation in either the NAc core or shell. After a stable baseline of sweet-fat diet consumption was established during limited access sessions (following 15–20 sessions), rats received 3 sessions of stimulation followed by 3 sham post-stimulation sessions. Animals then entered a 2 week washout phase to re-establish baseline prior to crossover and stimulation in the other target (Figure 1A).

## Data analysis

### Experiment 1 data analysis

In order to evaluate the effect of DBS in Experiment 1, we defined a meaningful DBS response as any change in consumption that exceeded 2 standard deviations of baseline consumption. To calculate the standard deviation of consumption, we pooled baseline binge eating data from multiple cohorts to characterize variation in baseline binge size within the population (36 rats, 3 baseline sessions per rat, 108 total baseline observations). The data came from all of the animals in this study, a previously published study (26), and unpublished data. Each observation was recorded as the percent change from that rats average baseline binge size. This “normalized variance” was done to account for the known variation between animals in their average binge size at baseline. This session to session normalized variation in binge size was found to be normally distributed, centered at 0% change with a standard deviation of 13% (Figure 1B). Thus, for Experiment 1, if an animal's binge size during a stimulation session was greater or less than 26% (2 standard deviations) of its average baseline binge size it was considered a meaningful change induced by stimulation.

### Experiment 2 data analysis

#### Group-based analysis

We used repeated measures analysis of variance (RMANOVA) and included 3 sessions of baseline, stimulation and post-stimulation data from each animal. Each stimulation target was analyzed independently, as there were no significant differences in binge size between the baseline periods on either side of the crossover. Session number (1–3) and session type (baseline, stimulation, and post-stimulation) were assumed to be categorical variables. When the analysis indicated that differences existed between session types, *post-hoc* pair-wise comparisons between groups were made using the Bonferroni method to correct for multiple comparisons.

#### Individual-based analysis

Individual rats were classified as either non-responders [NR] or responders [R] to stimulation at each target based on the criteria used in Experiment 1 (greater than a 2 standard deviation (26%) change in binge size from each animal's baseline average) and this change had to be observed in all three stimulation sessions for a given target.

## Experiment 3 – behavioral and electrical characterization (without stimulation)

All rats from Experiment 2 (*N* = 9) and those rats from Experiment 1 tested with the stimulation parameters chosen for Experiment 2 in both targets (*N* = 3) were included in Experiment 3 (*N* = 12). These animals underwent subsequent behavioral and electrophysiological characterization starting 2 weeks after the conclusion of Experiment 1 or 2. All rats underwent behavioral testing followed by another 2 week washout and then electrophysiological characterization of each stimulation site, but all without stimulation (Figure 1A).

### Reward-related behavior (order of testing)

To determine if variation in reward-related behavior could capture the underlying network differences that may be responsible for the variation in DBS outcomes, 3 reward-related behaviors were assessed. The behaviors were selected because they could be succinctly implemented and had previous evidence supporting the involvement of the NAc. These behaviors relate to binge eating because of the overlapping involvement of the NAc within the networks that underpin them. Behavioral outcomes were compared between NR and R groups for each DBS target using a two-way *t*-test. A significance threshold of *p* < 0.05 was used to screen for a potential relationship between reward-related behavior and stimulation outcomes.

#### Increased sweet-fat diet intake with food deprivation (1)

Food deprivation (24 h) was used to push the energy homeostasis system toward an orexigenic state. Individual variation in the resultant changes in binge size from baseline was measured and provided a reflection of the interplay between the systems controlling energy homeostasis and those regulating motivated behavior. Thus, the primary outcome was the percent change in binge size from each rat's baseline average to that observed following food deprivation.

#### Locomotor response to novelty (2)

Locomotor response to novelty was chosen because of previous correlations between variation in this behavior (high and low responders) and a sensation-seeking behavioral phenotype linked to a higher risk for developing disorders of appetitive behavior (33, 34). Briefly, rats were placed in a 1.5 × 3 ft black plastic chamber that was novel to the animal and allowed to freely explore for 50 min while video was recorded. Video files were analyzed offline using automated contrast-based tracking (Cineplex software, *Plexon, Plano, TX)* to calculate the distance traveled (primary outcome).

#### Conditioned place preference (CPP) (3)

CPP was assessed due to the known involvement of the NAc in CPP (35). We used an established 2-chamber biased design paradigm, pairing the sweet-fat food with the individual animal's non-preferred chamber and regular house chow with the preferred chamber (30 min pairing, 1 pairing per day, alternating between the 2 chambers for 4 days) (36, 37). Baseline and test sessions (15 min) were video recorded and automatically scored using contrast-based tracking to assess time spent in each chamber. The primary outcome was the change in the percentage of time spent in the initially non-preferred chamber (paired with sweet-fat diet).

### Local field potential (LFP) recording

We recorded local field potential (LFP) activity bilaterally from the NAc core and shell of each animal to assess whether variation of intrinsic network characteristics in the absence of stimulation could classify stimulation outcomes. Rats were tethered in a neutral chamber through a commutator to a Plexon data acquisition system while time-synchronized video images were recorded (*Plexon, Plano, Tx*) for offline analysis. Using the video images, rest intervals were manually identified as extended periods of inactivity, and only recordings from these intervals were used in the analysis. We used well-established frequency ranges from the rodent literature and standard LFP signal processing to characterize the power spectral densities (PSDs) within, and coherence between brain regions (bilateral NAc core and shell) for each animal using custom code written using Matlab R2015b (38–40) (Supplemental Methods). Each rat recording session produced 60 LFP features: 24 measures of power (6 frequency bands × 4 brain locations) and 36 measures of coherence (6 frequency bands × 6 possible location pairs, **Figures 5A,B**). We obtained two recordings from each animal that were separated in time by between 2 and 71 days to control for potential day to day variation in LFPs.

### Linking ventral striatal activity to stimulation outcomes

We built models using ventral striatal LFPs to classify stimulation outcomes and identify the optimal target for stimulation within an individual. As there were many more predictor variables than number of animals, we employed a machine learning approach to determine if there was information within the LFP signals that could classify stimulation outcomes. We used a penalized regression method, lasso, to reduce the dimensionality of the predictor variable set by removing LFP features that contained no information or redundant information and extracted the smallest combination of LFP features that most accurately described the observed variation in stimulation outcomes. The Matlab package *Glmnet* was used to implement the lasso method using a 4-fold cross-validation scheme with 100 repetitions for each model (Core R vs. NR, Shell R vs. NR, and Core vs. Shell). For the Core vs. Shell model, each animal's optimal stimulation target was defined as the stimulation target that produced the largest average reduction in binge size (rats without a significant reduction were excluded). The accuracy of the models is reported as the average cross-validated accuracy. In order to determine if the achieved accuracies were meaningfully better then chance, the entire process described above was repeated for ten random permutations of the data for each model type. The permutations randomized the relationship between the binary stimulation outcomes (*R* = 1, *NR* = 0) or optimal target assignment (Core = 1, Shell = 0) with the individual rat LFP feature sets to maintain the overall structure of the data, but permute the relationship of dependent to independent variables. The distribution of accuracies from the observed data was compared to the distribution from the permuted data using the Mann-Whitney *U* test, and the *U* test statistic was converted into a Cohen's d effect size.

If the lasso indicated that information existed in the LFP signal, a subsequent investigation of each LFP feature was carried out to determine which features contained the most information. For this, logistic regressions were implemented using the Matlab function *fitglm* to build models to classify: (1) core responses; (2) shell responses; or (3) core or shell as the best stimulation target for each animal. For the logistic models, an exhaustive leave-one-out, cross-validation was used to obtain a distribution of accuracies, and the mean accuracy from these distributions is reported in Table 1 for the top 5 LFP features from each model type.

**Table 1:** Top 5 LFP Features for Each Model Type.

**Logistic**			**Lasso**		
	**Features**	**% Accuracy**	**R**	**Features**	**% Survival**
**CORE**					
↑	Cslcl Δ	81	↑	Cclcr hγ	98
↑	Cslcr Δ	76	↓	Cclcr lγ	88
↑	Psr Δ	70	↑	Cclsr θ	86
↑	Cclcr hγ	70	↓	Psl Δ	76
↓	Cclcr Δ	68	↓	Psr θ	74
**SHELL**					
↓	Cclcr Δ	73	↑	Pcr Δ	86
↑	Pcr Δ	71	↓	Cslsr θ	85
↓	Cclsr Δ	70	↓	Pcr α	81
↓	Cslsr θ	70	↑	Psl β	58
↑	Cslcl lγ	68	↑	Cclcr β	53
**CORE VS. SHELL**					
↑	Cclcr hγ	79	↑	Cclsr Δ	60
↓	Pcr Δ	78	↑	Cslsr θ	55
↓	Cclcr β	77	↓	Psl θ	51
↑	Cclcr Δ	76	↓	Pcr Δ	49
↑	Csrcr θ	75	↑	Cslsr lγ	12

*The top 5 local field potential features used in single predictor (logistic) and multi-predictor (lasso) models of NAc core and shell stimulation outcomes. Features are described by location (CL, Core Left; CR, Core Right; SL, Shell Left; SR, Shell Right) and frequency band (delta -Δ, theta -θ, alpha -α, beta -β, low gamma -lγ, and high gamma -hγ). Power features are represented with location and frequency band (e.g., PSR Δ) and coherence features are represented with location pairs and frequency band (e.g., CSLCL Δ). Logistic features were ranked by the average % accuracy of the single variable logistic model using leave one out cross-validation. Lasso features were ranked by how frequently they were used in the lasso models from 100 iterations of cross-validation (% survival). Arrows to the left of the LFP feature indicate whether higher (up) or lower (down) LFP feature values increased the probability of a DBS response (R), or in the Core vs. Shell model the direction that increased the likelihood that Core is the better target for that animal*.

### Verification of electrode placement

At the conclusion of all experiments, rats were euthanized, and the brains were removed, prepared for cryostat sectioning, mounted on slides, and stained (thionine) for histological analysis of electrode placement (26). All animals included in the results had electrodes located within the target structures (**Figure 4C**).

## Results

### Experiment 1 – identifying optimal stimulation parameters

**Figure 2:**
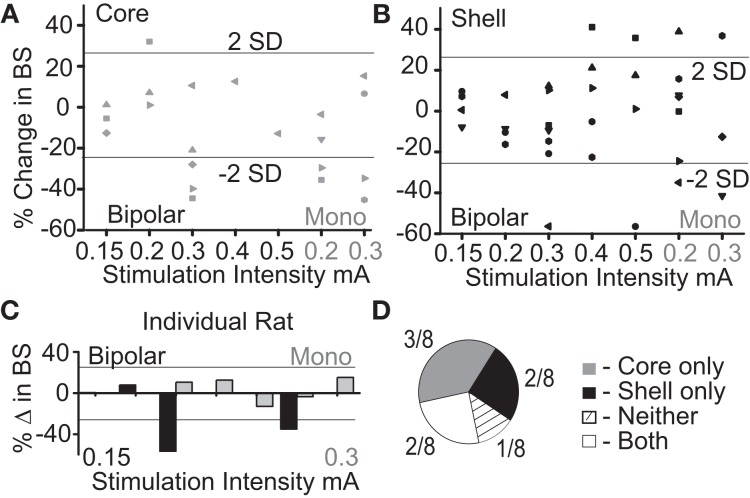
Optimal stimulation parameters were identified that could reduce binge size (BS) using the electrode arrays targeting the NAc core and shell. **(A)** Titration of stimulation parameters in NAc core reveals bipolar 300 μA and monopolar 200 μA are both effective and roughly equivalent. Bipolar (black) and monopolar (Mono, gray) stimulation configurations with corresponding current intensities shown on x-axis. **(B)** Titration of stimulation parameters in NAc shell showing similar effective parameters. **(C)** Example of a single rat's stimulation response profile illustrating a shell only responder (core-gray; shell-black). Horizontal lines illustrate ± 2 standard deviations (± 26%). **(D)** Distribution of stimulation response profiles for this cohort showing that 5/8 animals responded to only one of the two stimulation targets.

The purpose of this experiment was to determine what stimulation parameters demonstrated the capacity to reduce feeding behavior in either stimulation target (core or shell). Figure 2A summarizes the outcome of stimulation in the NAc core; significant reductions in food intake were observed with a bipolar configuration (300 μA) in 3/8 animals and with monopolar configuration (200–300 μA) in 4/8 animals. Figure 2B summarizes the outcomes of stimulation of the NAc shell in which significant reductions in food intake were observed in a subset of animals that received bipolar and monopolar stimulation. Interestingly, a subset of the shell-stimulated animals had significant increases in food intake at higher stimulation intensities. An example of an individual rat's food intake across tested stimulation parameters in the NAc core and shell is shown in Figure 2C. There were significant reductions in food intake during stimulation in the NAc shell at bipolar 300 μA and monopolar 200 μA with no significant food intake changes with core stimulation. Figure 2D illustrates the entire cohort's individual response profiles.

As demonstrated by the example rat, many animals responded to stimulation in only one of the two NAc sub-regions, despite testing across a range of stimulation parameters. Overall, this cohort of animals helped us identify a stimulation configuration (*[monopolar]* and parameters *[130 Hz, 90* μ*s pulse width, and 200* μ*A]*) for the custom arrays that was capable of decreasing food intake when either the NAc core or shell was targeted.

### Experiment 2 – testing NAc core vs. shell stimulation using optimized stimulation parameters

**Figure 3:**
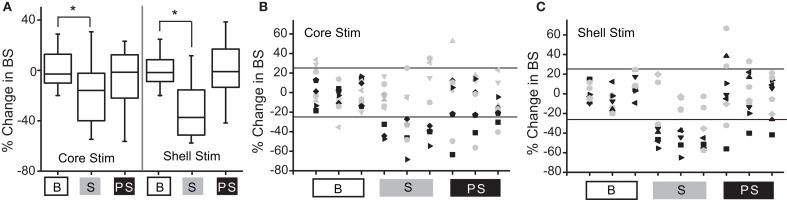
Deep brain stimulation targeted to either the NAc core or shell produces significant reductions in binge size using group-based analysis but with clear individual responders and non-responders. **(A)** Group-based analysis (RMANOVA) with *post-hoc* evaluation revealed a significant difference between baseline (B) and stimulation (S) sessions but not between baseline and post-stimulation (PS) sessions with either core or shell targeted stimulation (^*^p 0.05, boxplots-95% CI). **(B)** Individual rat responses to core stimulation with responders (black, 4/9) and non-responders (gray, 5/9). Horizontal lines illustrate ± 2 standard deviations (± 26%). **(C)** Individual rat responses to shell stimulation with responders (black, 5/9) and non-responders (gray, 4/9).

Figure 3A shows the population outcomes for this cohort (*N* = 9) using the same stimulation parameter in both the NAc core and shell. Using population statistics (RMANOVA), a main effect for session type (baseline, stimulation, post-stimulation) was observed in the shell stimulation set [*F*_(1, 8)_ = 8.171, *P* = 0.02] and in the core stimulation set [F_(1, 7)_ = 3.772, *P* = 0.05]. In order to determine which sessions were different, *post-hoc* pairwise comparisons with Bonferroni adjustment showed a significant difference between the baseline sessions and each stimulation session (*p* < 0.05), but not between the baseline sessions and the post-stimulation sessions.

To determine which rats responded to NAc core and shell stimulation, our *a priori* definition of responders and non-responders was used. The individual responses to NAc core and shell stimulation are shown in Figures 3B,C respectively, with significant individual responders shown in black and non-responders shown in gray. In this cohort, 3/9 rats responded only to shell stimulation, 2/9 rats responded only to core stimulation, 2/9 rats responded to either location and 2/9 did not respond to either location. Thus, 5/9 rats responded to stimulation in only one of the two targets. Overall (Experiment 1 and 2), 10/17 rats (~60%) responded to only one of the two stimulation targets, 4/17 responded to either target and 3/17 did not respond. These results highlight the potential need for individualized targeting of stimulation.

### Experiment 3 – behavioral and electrical characterization (without stimulation)

#### Relationship between stimulation outcomes and reward-related behavior

**Figure 4:**
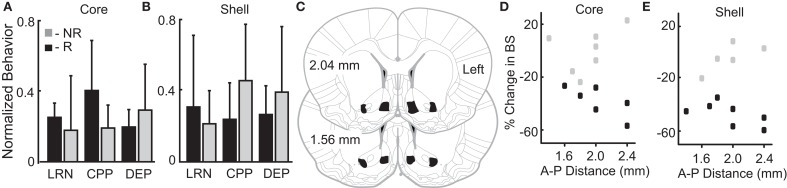
Variation in reward-related behavior and electrode location does not relate to stimulation outcomes. Normalized behavioral data grouped by core **(A)** and shell **(B)** DBS response type–responders (R; black) and non-responders (NR; gray). No significant differences were observed between R and NR groups for the following outcomes: (1) total distance traveled during locomotor response to novelty (LRN); (2) change in the percent of time spent in the initially non-preferred chamber during conditioned place preference (CPP); and (3) percentage increase in food intake after 24 h of food deprivation (DEP). **(C)** All rats included in the analysis had electrode locations within the bilateral NAc core and shell with electrodes localized within the black shapes collapsed onto two representative coronal sections. The largest variation in electrode positioning occurred along the anterior-posterior (A-P) dimension (1.4 to 2.4 mm anterior to bregma). No discernable relationship between electrode placement along the A-P axis in NAc core **(D)** or shell **(E)** corresponded to stimulation outcomes – responder (black) or non-responder (gray).

We theorized that innate variation in networks including the NAc core and shell could be a common source of variation underlying individual differences in reward-related behavior and stimulation outcomes. Thus, we examined a relationship between variation in reward-related tasks (reflecting differences in networks that include the NAc) and stimulation outcomes. The behavioral metrics of the 12 rats studied were grouped based on the rat's individual response to stimulation as defined previously (R-responder and NR-non-responder for each stimulation target). Differences between the R and NR groups were evaluated with *t*-tests. None of the behavioral measures differed as a function of the R/NR grouping for either stimulation site, core- (Figure 4A) or shell- (Figure 4B).

#### Relationship between stimulation outcomes and electrode localization

Figures 4C–E illustrates the relationship of anterior-posterior (A-P) position in the core (Figure 4D) and the shell (Figure 4E) and the corresponding stimulation outcomes (black-responders; gray-non-responders). Variation of electrode location within the A-P dimension displayed no discernable relationship with stimulation outcomes. It is important to note that previously published estimates of the effective electric field for similar stimulation parameters and type (monopolar) estimate a spherical shape with a radius ~0.5 mm or less (41, 42). This suggests that non-overlapping neural volumes were likely targeted given the distance between core and shell electrodes (~1.4 mm)—verified by histology.

#### Relationship between stimulation outcomes and local field potential activity

**Figure 5:**
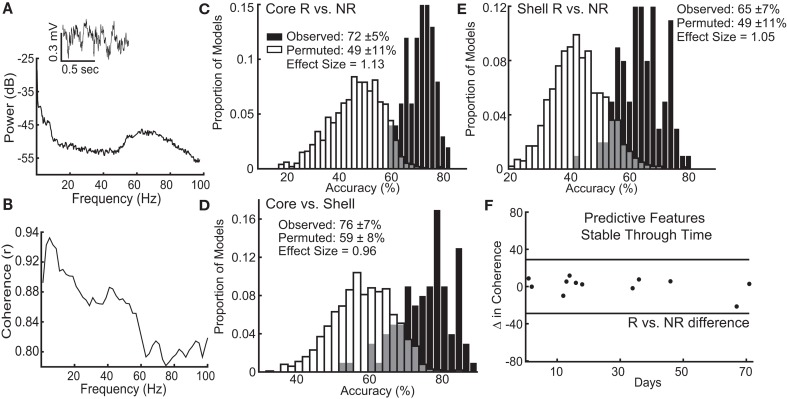
Local field potential (LFP) features recorded from ventral striatum can classify individual stimulation outcomes and are stable through time. **(A)** Inset of a raw LFP trace from the left NAc core with its corresponding power spectral density plot. **(B)** Corresponding coherence plot showing phase relationships across frequencies between the left NAc shell and right NAc core. The distribution of accuracies from classifying NAc core **(C)** and shell **(E)** stimulation responders (R) from non-responders (NR) using the observed data (black) and the permuted data (white) with mean accuracy ± standard deviation listed for each distribution. Effect sizes between observed and permuted distributions are also shown. **(D)** Distribution of accuracies classifying the optimal target for stimulation (core vs. shell) for each animal using the observed data (black) or the permuted data (white). **(F)** The difference in delta coherence (between the left NAc core and right NAc shell) from recording day T1 to T2 (up to 71 days apart) was smaller than the difference observed between the groups of animals that preferentially responded to core or shell.

The lasso used information contained within LFP features, existing at the stimulation sites when stimulation was not present, to determine which response group an animal belonged to with an average accuracy for core stimulation of 72% (standard deviation ± 5%), outperforming the models produced from random permutations of the data (49% accuracy ± 11%) with an effect size of 1.13 (Figure 5C). The lasso models classifying shell stimulation outcomes performed with an average accuracy of 65% (standard deviation ± 7%), outperforming the models produced from random permutations of the data (49% accuracy ± 11%) with an effect size of 1.05 (Figure 5E). Finally, each rat with a significant reduction in binge size was grouped by the target (NAc core or shell) that produced the largest average reduction in binge size across the three stimulation sessions. LFP features were able to match individual rats to the most effective target for stimulation using lasso with an average accuracy of 76% (standard deviation ± 7%) compared to 59% (standard deviation ± 8%) for the permuted data with an effect size of 0.96 (Figure 5D).

It is important to note that each rat had 2 LFP recording sessions separated by up to 71 days, and each recording session was separately incorporated into the model. Therefore, only LFP features that had stable differences between groups (e.g., R vs. NR) across time were selected and used by lasso. An example of one of the selected LFP features is shown in Figure 5F, which indicates that the feature varied less between day 1 and day 71 within each animal than it did between the responder and non-responder groups (Figure 5F–black horizontal lines). This finding indicates that the information about stimulation outcomes extracted from LFP signals was stable through time and raises the possibility that these differences exist prior to stimulation.

To determine which components of the LFP signal contained the most information about stimulation outcomes, each feature's performance in logistic models (% accuracy) was compared to how commonly those features were included in the (lasso) models (% survival). Table 1 lists the top 5 LFP features from the logistic and lasso models of core and shell stimulation outcomes (R vs. NR) and the classification of the optimal target for each animal (core vs. shell). This exploration revealed a predominance of delta band features in the logistic models that did not translate to survival in the lasso models suggesting that while delta features contained the most information about outcomes, this information was likely highly redundant as lasso removes features with redundant infomation. Thus, only one delta feature tended to be included in the lasso models. Arrows in the table indicate the directionality of the feature differences between groups.

## Discussion

These experiments demonstrate that deep brain stimulation of either the nucleus accumbens core or shell, regions with known differences in brain connectivity and distinct functional roles in appetitive behaviors, have a similar capacity to reduce “binge-like” feeding behavior. Experiment 1 demonstrated that despite titration across multiple stimulation parameters only subsets of animals showed significant changes in binge behavior with stimulation in either of the tested targets. Experiment 2 confirmed this finding and individual responses across the first two experiments illustrated that 66% of rats respond to DBS in only one of the two targets, supporting the likelihood that personalized target selection could improve treatment outcomes. Experiment 3 demonstrated that variation in stimulation outcomes could be, in part, explained by individual differences in recorded local field potential activity in the absence of stimulation using a machine learning-based approach (lasso). LFPs recorded from network nodes underlying appetitive behavior contained information about whether a given individual achieved a meaningful suppression of binge eating with stimulation. Most importantly, ventral striatal oscillations were also capable of classifying the most effective stimulation target for each individual, demonstrating the potential of using network activity under “resting,” unstimulated conditions to classify the optimal target for neuromodulation. However, it must be noted that these recordings and classifications were done post hoc, therefore it would be fruitful to verify these results in future work in which the recordings are done prior to stimulation.

The translational relevance of this work is supported by treatment outcome variability that has been previously observed in clinical studies of focal stimulation in disorders of appetitive behavior (6, 13, 43). As an example, in a study using repetitive transcranial magnetic stimulation of the medial prefrontal cortex for patients with binge eating, differences in cortical-striatal network activity were shown to correlate with responses to stimulation (27). Therefore, it is notable in this study that a large proportion of animals that failed to respond to stimulation in one brain target (NAc shell), responded to stimulation in an alternative target (NAc core). Further, results from this study suggest that network activity recorded without stimulation in the ventral striatum contains information that can classify the optimal target for stimulation on an individual basis. This finding suggests that even in this outbred rat model of binge eating, there may be individual differences in the networks perpetuating the behavioral expression of binge eating.

The assertion that variation exists across individuals in the specific cortical-striatal networks that underpin the expression of appetitive behavior is supported by a rich literature including the well characterized spectrum of goal-directed to habitual behavior (29, 44–46). Thus, the striatal sub-regions driving binge-like behavior could vary across individuals and impact which striatal target (NAc shell vs. core) is most likely to modulate binge behavior. Patients with binge eating have also been shown to display altered function in distinct networks including the reward/salience network (47–49) and/or the cortical control network (50–53) using non-invasive methods to assess network activity. Altered function of one of these networks may be enough to perpetuate binge eating (54), and our work in rats suggests that even within the ventral striatum, different sub-circuits (involving the NAc core or shell) may be underlying the perpetuation of binge eating across individuals. Both clinical and pre-clinical studies suggest that a single stimulation target may not have the capacity to reduce binge eating across all individuals, and our results suggest that measures of relevant network activity could guide the selection of an effective stimulation target for each individual.

To translate personalized targeting of neuromodulation-based treatments to patients, the relevant network activity would have to be measured prior to the intervention. This could be accomplished with the use of intracranial electrodes as is done prior to surgery for epilepsy or using a non-invasive approach (e.g., MRI-based). Thus, it is important to consider the relationship between information extracted from LFP oscillations recorded from depth electrodes reported in this study and non-invasive methods of measuring related network activity in patients. Our data suggest that inter-hemispheric coherence at low frequencies (delta and theta) may be a rich source of information that could be used to classify DBS outcomes. Previous work has established that a correlation exists between these LFP features and fMRI derived measures, including resting state functional connectivity (55–58). The work presented in this study supports the inclusion of the ventral striatum and interconnected cortical regions for future investigations that attempt to use brain activity to guide targeting of focal stimulation for binge eating and related disorders of appetitive behavior.

Overall this study was limited by small sample sizes, and although using a machine learning approach (lasso) mitigated the problem of having many more predictor variables than observations, a larger sample size would allow testing of the tuned models on naïve datasets and provide more power to relate variation in electrode location and behavior with stimulation outcomes. Future studies will incorporate pre-stimulation recordings in order to evaluate the capacity of network activity from treatment naïve animals to predict future treatment outcomes and optimal stimulation targets. The scope of information used to build our classification models could be expanded by increasing the number of recording sites to include additional regions in the distributed feeding circuit (e.g., hypothalamic/brainstem, medial prefrontal and orbitofrontal cortex). In particular, recording from cortical regions would have translational relevance to non-invasive clinical measures of brain activity (e.g., EEG) in addition to MRI derived features. Further, although it is possible that models using brain activity during the feeding behavior rather than rest may perform better, collecting brain data during binge eating in patients is much less feasible than collecting resting state data. We cannot rule out the possibility that variation in targeting within the NAc sub-regions also contributed to stimulation outcome variation. Inclusion of a female cohort would have increased the generalizability of this study as more women suffer from binge eating compared to men. Last, none of the reward-related behaviors tested in this study showed potential to classify stimulation outcomes, but alternative reward-related behaviors may better reflect the individual differences that may underlie the variation in stimulation outcomes (45, 59).

## Conclusion

For the treatment of many psychiatric disorders, as demonstrated here in a rat model of binge eating, a single target for neuromodulation-based treatment may not be effective across all individuals. Rather, an individualized treatment approach that uses network activity to guide the personalization of target selection could reduce current treatment outcome variability.

## Author contributions

WD contributed to all aspects of this project. LD performed the signal processing and computational analysis. JB and AS significantly contributed to data acquisition. JK performed statistical analysis and manuscript editing along with AG.

### Conflict of interest statement

By way of disclosure, over the past 3 years, AG received research grants from Alkermes, Novartis, and Janssen. He has served as an (uncompensated) consultant to Otsuka and Alkermes, and as a member of a Data Monitoring Board for Lilly. The remaining authors declare that the research was conducted in the absence of any commercial or financial relationships that could be construed as a potential conflict of interest.

## References

[1] Sturm V Lenartz D Koulousakis A Treuer H Herholz K Klein JC. The nucleus accumbens: a target for deep brain stimulation in obsessive-compulsive- and anxiety-disorders. J Chem Neuroanat. (2003) 26:293–9. 10.1016/j.jchemneu.2003.09.003 14729131

[2] Mayberg HS Lozano AM Voon V Mcneely HE Seminowicz D Hamani C. Deep brain stimulation for treatment-resistant depression. Neuron (2005) 45:651–60. 10.1016/j.neuron.2005.02.014 15748841

[3] Toft M Lilleeng B Ramm-Pettersen J Skogseid IM Gundersen V Gerdts R. Long-term efficacy and mortality in Parkinson's disease patients treated with subthalamic stimulation. Mov Disord. (2011) 26:1931–4. 10.1002/mds.23817 21656853

[4] Schlaepfer TE Cohen MX Frick C Kosel M Brodesser D Axmacher N. Deep brain stimulation to reward circuitry alleviates anhedonia in refractory major depression. Neuropsychopharmacology (2008) 33:368–77. 10.1038/sj.npp.1301408 17429407

[5] Ahmari SE Dougherty DD. (2015). Dissecting OCD circuits: from animal models to targeted treatments. Depress Anxiety. 32:550–62. 10.1002/da.22367 25952989 PMC4515165

[6] Deeb W Giordano JJ Rossi PJ Mogilner AY Gunduz A Judy JW. Proceedings of the fourth annual deep brain stimulation think tank: a review of emerging issues and technologies. Front Integr Neurosci. (2016) 10:38. 10.3389/fnint.2016.00038 27920671 PMC5119052

[7] Riva-Posse P Choi KS Holtzheimer PE Mcintyre CC Gross RE Chaturvedi A. Defining critical white matter pathways mediating successful subcallosal cingulate deep brain stimulation for treatment-resistant depression. Biol Psychiatry (2014) 76:963–9. 10.1016/j.biopsych.2014.03.029 24832866 PMC4487804

[8] Smart OL Tiruvadi VR Mayberg HS. Multimodal approaches to define network oscillations in depression. Biol Psychiatry (2015) 77:1061–70. 10.1016/j.biopsych.2015.01.002 25681871 PMC5826645

[9] Filkowski MM Mayberg HS Holtzheimer PE. Considering eligibility for studies of deep brain stimulation for treatment-resistant depression: insights from a clinical trial in unipolar and bipolar depression. J ECT (2016) 32:122–6. 10.1097/YCT.0000000000000281 26479487 PMC4834065

[10] Valencia-Alfonso CE Luigjes J Smolders R Cohen MX Levar N Mazaheri A. Effective deep brain stimulation in heroin addiction: a case report with complementary intracranial electroencephalogram. Biol Psychiatry (2012) 71:e35–7. 10.1016/j.biopsych.2011.12.013 22281120

[11] Whiting DM Tomycz ND Bailes J De Jonge L Lecoultre V Wilent B. Lateral hypothalamic area deep brain stimulation for refractory obesity: a pilot study with preliminary data on safety, body weight, and energy metabolism. J Neurosurg. (2013) 119:56–63. 10.3171/2013.2.JNS12903 23560573 PMC5666570

[12] Nangunoori RK Tomycz ND Oh MY Whiting DM. Deep brain stimulation for obesity: from a theoretical framework to practical application. Neural Plast. (2016) 2016:7971460. 10.1155/2016/7971460 26819774 PMC4706960

[13] Terraneo A Leggio L Saladini M Ermani M Bonci A Gallimberti L. Transcranial magnetic stimulation of dorsolateral prefrontal cortex reduces cocaine use: a pilot study. Eur Neuropsychopharmacol. (2016) 26:37–44. 10.1016/j.euroneuro.2015.11.011 26655188 PMC9379076

[14] Spagnolo PA Goldman D. (2017). Neuromodulation interventions for addictive disorders: challenges, promise, and roadmap for future research. Brain 140:1183–1203. 10.1093/brain/aww284 28082299 PMC6059187

[15] Luigjes J Van Den Brink W Feenstra M Van Den Munckhof P Schuurman PR Schippers R. Deep brain stimulation in addiction: a review of potential brain targets. Mol Psychiatry (2012) 17:572–83. 10.1038/mp.2011.114 21931318

[16] Guo L Zhou H Wang R Xu J Zhou W Zhang F. DBS of nucleus accumbens on heroin seeking behaviors in self-administering rats. Drug Alcohol Depend. (2013) 129:70–81. 10.1016/j.drugalcdep.2012.09.012 23062870

[17] Pierce RC Vassoler FM. Deep brain stimulation for the treatment of addiction: basic and clinical studies and potential mechanisms of action. Psychopharmacology (2013) 229:487–91. 10.1007/s00213-013-3214-6 23907650 PMC3786680

[18] Corwin RL. Binge-type eating induced by limited access in rats does not require energy restriction on the previous day. Appetite (2004) 42:139–42. 10.1016/j.appet.2003.08.010 15010177

[19] Corwin RL Buda-Levin A. Behavioral models of binge-type eating. Physiol Behav. (2004) 82:123–30. 10.1016/j.physbeh.2004.04.036 15234600

[20] Berner LA Avena NM Hoebel BG. Bingeing, self-restriction, and increased body weight in rats with limited access to a sweet-fat diet. Obesity (2008) 16:1998–2002. 10.1038/oby.2008.328 19186326

[21] Oswald KD Murdaugh DL King VL Boggiano MM. Motivation for palatable food despite consequences in an animal model of binge eating. Int J Eat Disord. (2011) 44:203–11. 10.1002/eat.20808 20186718 PMC2941549

[22] Heal DJ Goddard S Brammer RJ Hutson PH Vickers SP. Lisdexamfetamine reduces the compulsive and perseverative behaviour of binge-eating rats in a novel food reward/punished responding conflict model. J Psychopharmacol. (2016) 30:662–75. 10.1177/0269881116647506 27170676

[23] Vickers SP Goddard S Brammer RJ Hutson PH Heal DJ. Investigation of impulsivity in binge-eating rats in a delay-discounting task and its prevention by the d-amphetamine prodrug, lisdexamfetamine. J Psychopharmacol. (2017) 31:784–97. 10.1177/0269881117691672 28372478

[24] Vickers SP Hackett D Murray F Hutson PH Heal DJ. Effects of lisdexamfetamine in a rat model of binge-eating. J Psychopharmacol. (2015) 29:1290–307. 10.1177/0269881115615107 26589243

[25] Mcelroy SL Hudson J Ferreira-Cornwell MC Radewonuk J Whitaker T Gasior M. Lisdexamfetamine dimesylate for adults with moderate to severe binge eating disorder: results of two pivotal phase 3 randomized controlled trials. Neuropsychopharmacology (2016) 41:1251–60. 10.1038/npp.2015.275 26346638 PMC4793109

[26] Doucette WT Khokhar JY Green AI. Nucleus accumbens deep brain stimulation in a rat model of binge eating. Transl Psychiatry (2015) 5:e695. 10.1038/tp.2015.197 26670280 PMC5068592

[27] Dunlop K Woodside B Lam E Olmsted M Colton P Giacobbe P. Increases in frontostriatal connectivity are associated with response to dorsomedial repetitive transcranial magnetic stimulation in refractory binge/purge behaviors. Neuroimage Clin. (2015) 8:611–8. 10.1016/j.nicl.2015.06.008 26199873 PMC4506986

[28] Burton AC Nakamura K Roesch MR. (2014). From ventral-medial to dorsal-lateral striatum: neural correlates of reward-guided decision-making. Neurobiol Learn Mem. 117:51–9 10.1016/j.nlm.2014.05.003 24858182 PMC4240773

[29] Heilbronner SR Rodriguez-Romaguera J Quirk GJ Groenewegen HJ Haber SN. Circuit-based corticostriatal homologies between rat and primate. Biol Psychiatry (2016) 80:509–21. 10.1016/j.biopsych.2016.05.012 27450032 PMC5438202

[30] Halpern CH Tekriwal A Santollo J Keating JG Wolf JA Daniels D. Amelioration of binge eating by nucleus accumbens shell deep brain stimulation in mice involves D2 receptor modulation. J Neurosci. (2013) 33:7122–9. 10.1523/JNEUROSCI.3237-12.2013 23616522 PMC3703148

[31] Wu H Miller KJ Blumenfeld Z Williams NR Ravikumar VK Lee KE. (2017). Closing the loop on impulsivity via nucleus accumbens delta-band activity in mice and man. Proc Natl Acad Sci USA. 10.1073/pnas.1712214114 29255043 PMC5776799

[32] Kuncel AM Grill WM. Selection of stimulus parameters for deep brain stimulation. Clin Neurophysiol. (2004) 115:2431–41. 10.1016/j.clinph.2004.05.031 15465430

[33] Piazza PV Deminiere JM Le Moal M Simon H. Factors that predict individual vulnerability to amphetamine self-administration. Science (1989) 245:1511–3. 10.1126/science.2781295 2781295

[34] Belin D Mar AC Dalley JW Robbins TW Everitt BJ. High impulsivity predicts the switch to compulsive cocaine-taking. Science (2008) 320:1352–5. 10.1126/science.1158136 18535246 PMC2478705

[35] Tzschentke TM. Measuring reward with the conditioned place preference (CPP) paradigm: update of the last decade. Addict Biol. (2007) 12:227–462. 10.1111/j.1369-1600.2007.00070.x 17678505

[36] Calcagnetti DJ Schechter MD. Extinction of cocaine-induced place approach in rats: a validation of the “biased” conditioning procedure. Brain Res Bull. (1993) 30:695–700. 10.1016/0361-9230(93)90102-H 8457916

[37] Valjent E Corbille AG Bertran-Gonzalez J Herve D Girault JA. Inhibition of ERK pathway or protein synthesis during reexposure to drugs of abuse erases previously learned place preference. Proc Natl Acad Sci USA. (2006) 103:2932–7. 10.1073/pnas.0511030103 16473939 PMC1413817

[38] Cohen MX Axmacher N Lenartz D Elger CE Sturm V Schlaepfer TE. Nuclei accumbens phase synchrony predicts decision-making reversals following negative feedback. J Neurosci. (2009) 29:7591–8. 10.1523/JNEUROSCI.5335-08.2009 19515927 PMC2725399

[39] Mccracken CB Grace AA. Nucleus accumbens deep brain stimulation produces region-specific alterations in local field potential oscillations and evoked responses *in vivo*. J Neurosci. (2009) 29:5354–63. 10.1523/JNEUROSCI.0131-09.2009 19386932 PMC2873195

[40] Catanese J Carmichael JE Van Der Meer MA. Low- and high-gamma oscillations deviate in opposite directions from zero-phase synchrony in the limbic corticostriatal loop. J Neurophysiol. (2016) 116, 5–17. 10.1152/jn.00914.2015 26961106 PMC4961757

[41] Arcot Desai S Gutekunst CA Potter SM Gross RE. Deep brain stimulation macroelectrodes compared to multiple microelectrodes in rat hippocampus. Front Neuroeng. (2014) 7:16. 10.3389/fneng.2014.00016 24971060 PMC4054883

[42] Hamani C Amorim BO Wheeler AL Diwan M Driesslein K Covolan L. Deep brain stimulation in rats: different targets induce similar antidepressant-like effects but influence different circuits. Neurobiol Dis. (2014) 71:205–14. 10.1016/j.nbd.2014.08.007 25131446 PMC5756089

[43] Azevedo CA Mammis A. (2018). Neuromodulation therapies for alcohol addiction: a literature review. Neuromodulation. 21:144–148. 10.1111/ner.12548 28055126

[44] Balleine BW O'doherty JP. Human and rodent homologies in action control: corticostriatal determinants of goal-directed and habitual action. Neuropsychopharmacology (2010) 35:48–69. 10.1038/npp.2009.131 19776734 PMC3055420

[45] Robinson TE Yager LM Cogan ES Saunders BT. (2014). On the motivational properties of reward cues: individual differences. Neuropharmacology 76(Pt B):450–9. 10.1016/j.neuropharm.2013.05.040 23748094 PMC3796005

[46] Voon V Reiter A Sebold M Groman S. Model-based control in dimensional psychiatry. Biol Psychiatry (2017) 82:391–400. 10.1016/j.biopsych.2017.04.006 28599832

[47] Svaldi J Tuschen-Caffier B Peyk P Blechert J. Information processing of food pictures in binge eating disorder. Appetite (2010) 55:685–94. 10.1016/j.appet.2010.10.002 20946926

[48] Michaelides M Thanos PK Volkow ND Wang GJ. Dopamine-related frontostriatal abnormalities in obesity and binge-eating disorder: emerging evidence for developmental psychopathology. Int Rev Psychiatry (2012) 24:211–8. 10.3109/09540261.2012.679918 22724642 PMC4827426

[49] Balodis IM Kober H Worhunsky PD White MA Stevens MC Pearlson GD. Monetary reward processing in obese individuals with and without binge eating disorder. Biol Psychiatry (2013) 73:877–86. 10.1016/j.biopsych.2013.01.014 23462319 PMC3686098

[50] Schienle A Schafer A Hermann A Vaitl D. Binge-eating disorder: reward sensitivity and brain activation to images of food. Biol Psychiatry (2009) 65:654–61. 10.1016/j.biopsych.2008.09.028 18996508

[51] Tammela LI Paakkonen A Karhunen LJ Karhu J Uusitupa MI Kuikka JT. Brain electrical activity during food presentation in obese binge-eating women. Clin Physiol Funct Imaging (2010) 30:135–40. 10.1111/j.1475-097X.2009.00916.x 20095978

[52] Hege MA Stingl KT Kullmann S Schag K Giel KE Zipfel S. Attentional impulsivity in binge eating disorder modulates response inhibition performance and frontal brain networks. Int J Obes. (2015) 39:353–60. 10.1038/ijo.2014.99 24909828

[53] Imperatori C Fabbricatore M Innamorati M Farina B Quintiliani MI Lamis DA. Modification of EEG functional connectivity and EEG power spectra in overweight and obese patients with food addiction: an eLORETA study. Brain Imaging Behav. (2015) 9:703–16. 10.1007/s11682-014-9324-x 25332109

[54] Dunlop KA Woodside B Downar J. Targeting neural endophenotypes of eating disorders with non-invasive brain stimulation. Front Neurosci. (2016) 10:30. 10.3389/fnins.2016.00030 26909013 PMC4754427

[55] Logothetis NK Pauls J Augath M Trinath T Oeltermann A. Neurophysiological investigation of the basis of the fMRI signal. Nature (2001) 412:150–7. 10.1038/35084005 11449264

[56] Wang L Saalmann YB Pinsk MA Arcaro MJ Kastner S. Electrophysiological low-frequency coherence and cross-frequency coupling contribute to BOLD connectivity. Neuron (2012) 76:1010–20. 10.1016/j.neuron.2012.09.033 23217748 PMC3531830

[57] Murta T Leite M Carmichael DW Figueiredo P Lemieux L. Electrophysiological correlates of the BOLD signal for EEG-informed fMRI. Hum Brain Mapp. (2015) 36:391–414. 10.1002/hbm.22623 25277370 PMC4280889

[58] Jaime S Gu H Sadacca BF Stein EA Cavazos JE Yang Y. (2017). Delta rhythm orchestrates the neural activity underlying the resting state BOLD signal via phase-amplitude coupling. Cereb Cortex 1–15. 10.1093/cercor/bhx310 29161352 PMC6490973

[59] Singer BF Guptaroy B Austin CJ Wohl I Lovic V Seiler JL. Individual variation in incentive salience attribution and accumbens dopamine transporter expression and function. Eur J Neurosci. (2016) 43:662–70. 10.1111/ejn.13134 26613374 PMC4783234

