# Supplementary material for: Machine Learning Based Classification of Deep Brain Stimulation Outcomes in a Rat Model of Binge Eating Using Ventral Striatal Oscillations
Source: Front Psychiatry. 2018 Aug 3;9:336. doi: 10.3389/fpsyt.2018.00336 (PMC6085408; doi:10.3389/fpsyt.2018.00336)
Supplement: Supplementary file 1 [file Data_Sheet_1.PDF]

## Supplemental Material

### Supplemental Methods

*Electrode design for experiment 1*

The electrodes were built using a 30 gauge polyimide cannula with one insulated 50μm wire stripped of insulation for the terminal 2 mm terminating flush with the end of each cannula and a second 75 μm wire passing through the cannula and extending 1 mm beyond the end of the cannula. This design allowed for titration of stimulation parameters from 150 to 500 μA in a bipolar configuration (between the two wires in each cannula) or a monopolar configuration (between the extending 75 μm wire and a skull screw over lambda). In some cases the compliance voltage of the stimulator prevented titration to maximal current intensities and in one case stimulation above 300 μA in bipolar configuration led to a seizure, thereby limiting titration in that animal.

*Data acquisition and signal processing details*

All of the following electrophysiological processing steps were done to each individual file. A fourth order Chebychev type I notch filter centered at 60 Hz was applied to all of the data to account for 60 Hz line noise. The data was then downsampled by a factor of five from 2 kHz to 400 Hz. A threshold of ± 2 mV was used to identify noise artifacts and remove data using intervals 12.5 milliseconds before and 40 seconds after the artifacts. The resting data were extracted using the event markers placed manually in the video (scoring). To capture the power and coherence dynamics of the signal we only used behavioral epochs that were at least 5 seconds long. For epochs that were longer than 5 seconds, we segmented them into 5 second sections removing the remainder to keep all of the data continuous over the same amount of time.

Power spectral densities (PSDs) were computed using MATLAB's pwelch function using a 1.6 second Hamming window with 50% overlap. The PSDs for each 5 second segment were then

averaged together to get a single representative PSD for the rest behavior. Total power (dB) per frequency range was calculated using the following ranges: delta (Δ) = 1-4 Hz, theta (θ) = 5-10 Hz, alpha (α) = 11-14 Hz, beta (β) = 15-30 Hz, low gamma (lγ) = 45-65 Hz, and high gamma (hγ) 70-90 Hz (Cohen *et al.* 2009; Catanese *et al.* 2016; McCracken and Grace 2009). To account for the 60 Hz notch filter, power values of frequencies from 59 to 61 Hz were not included in the sum. The power per frequency band was then normalized as a percent of the average total power of the signal from 1 to 90 Hz (beginning of Δ to end of high γ).

Coherence was computed using *mscohere* with a 1.3 second sliding Hamming window with 50% overlap. The average coherence between each pair of channels from 1 to 90 Hz (excluding values corresponding to the notch filter) was used to normalize the average coherence of each frequency band within that channel pair.

*Statistical analysis*

Raw data was normalized as z-scores so that power and coherence were at the same scale. The MATLAB package *Glmnet* was used to tune and implement the lasso. The lasso penalized regression method uses a penalty term, $L_1$-norm, weighted by the parameter λ that sets coefficients to zero thus reducing the dimensionality of the model produced with the simplest models produced with large λ. The λ parameter was tuned through 100 iterations of 4-fold cross-validation using *cvglmnet* and for each iteration we extracted the average cross-validated error over all λ and the value of λ that was one standard deviation away from the λ with the least error. The λ that was +1 S.E. away from the minimum was selected in order to retain the least complex models (larger L1 penalty term shrinking more βs to zero) while not significantly increasing the model's error Although this repeated cross-validation scheme gives a precise estimate of accuracy (decreases variance) it does not overcome any potential bias in the estimate due to the lack of a naïve dataset [1, 2].

To test if the results of the lasso were entirely due to our small sample size and lack of a naïve dataset, the entire procedure described above was run 10 times using permuted data in which the relationship between outcome and their explanatory variables were shuffled according to Monte Carlo sampling. From this, a distribution of 1,000 accuracies was generated per model. Effect size was calculated by converting the *U* statistic from a Mann-Whitney *U* test into an r value and then into a Cohen's d (Rosenthal 1984).

The survival rate for each variable was calculated using the number of non-zero β coefficients, i.e. the percent of all 100 iterations that the β coefficient was not zero. We used a cutoff of 40% survival (eight times more stringent than the 5% cutoff applied by Ahn *et al.*, 2016) to populate network models [3].

**Supplemental Results**

**Supplemental Figure Legend**

**Figure S1.** No effect of stimulation on chow consumption and no relationship of shell stimulation outcomes to variation in tested reward related behaviors. **A.** Normalized behavioral outcomes (LRN - locomotor response to novelty; CPP - conditioned place preference; and Binge - binge size increase with 24 hours of food deprivation) divided into shell stimulation outcome groups (R-responder--black and NR-non-responder--grey). Bars depict group means +/- 1 standard deviation. There were no significant differences between R and NR groups for any of the behaviors (t-test). **B.** Stimulation to either the NAc core or shell did not produce any significant variation in chow consumption from baseline sessions to stimulation sessions (Stim) or post-stimulation sessions (Post Stim).

**Supplemental References**

[1] Blockeel C, Van Vaerenbergh I, Fatemi HM, Van Lommel L, Devroey P, Bourgain C. Gene expression profile in the endometrium on the day of oocyte retrieval after ovarian stimulation with low-dose hCG in the follicular phase. Mol Hum Reprod 2011;17(1):33-41.

[2] Zou H, Hastie T. Regularization and variable selection via the elastic net (vol B 67, pg 301, 2005). Journal of the Royal Statistical Society Series B-Statistical Methodology 2005;67:768-.
[3] Ahn WY, Ramesh D, Moeller FG, Vassileva J. Utility of Machine-Learning Approaches to Identify Behavioral Markers for Substance Use Disorders: Impulsivity Dimensions as Predictors of Current Cocaine Dependence. Front Psychiatry 2016;7:34.
