# Supplementary figures and images for: Machine Learning Based Classification of Deep Brain Stimulation Outcomes in a Rat Model of Binge Eating Using Ventral Striatal Oscillations
Source: Front Psychiatry. 2018 Aug 3;9:336. doi: 10.3389/fpsyt.2018.00336 (PMC6085408; doi:10.3389/fpsyt.2018.00336)

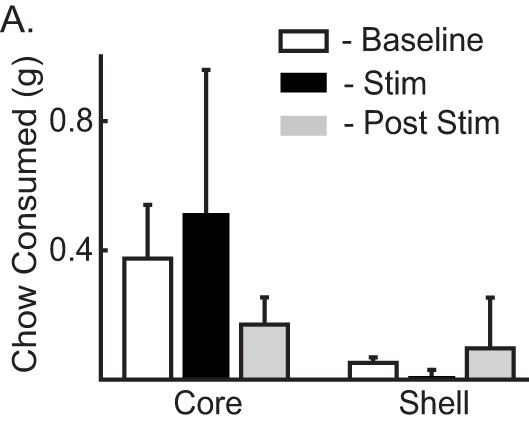

Supplement: Supplementary file 2 [file Image_1.jpg]
